# Clinical Experience with High-Dose Polymyxin B against Carbapenem-Resistant Gram-Negative Bacterial Infections—A Cohort Study

**DOI:** 10.3390/antibiotics9080451

**Published:** 2020-07-27

**Authors:** Yiying Cai, Hui Leck, Ray W. Tan, Jocelyn Q. Teo, Tze-Peng Lim, Winnie Lee, Maciej Piotr Chlebicki, Andrea L. Kwa

**Affiliations:** 1 Department of Pharmacy, Singapore General Hospital, Singapore 169608, Singapore; cai.yiying@sgh.com.sg (Y.C.); LECK_Hui@moh.gov.sg (H.L.); jocelyn.teo.q.m@sgh.com.sg (J.Q.T.); lim.tze.peng@sgh.com.sg (T.-P.L.); winnie.lee.h.l@sgh.com.sg (W.L.); 2 Department of Pharmacy, National University of Singapore, Singapore 117559, Singapore; ray.tan@parkwaypantai.com; 3 Saw Swee Hock School of Public Health, National University of Singapore and National University Health Systems, Singapore 117549, Singapore; 4 Academic Clinical Programme (Pathology), SingHealth Duke-NUS, Singapore 169857, Singapore; 5 Academic Clinical Programme (Medicine), SingHealth Duke-NUS, Singapore 169857, Singapore; 6 Department. of Infectious Diseases, Singapore General Hospital, Singapore 169608, Singapore; piotr.chlebicki@singhealth.com.sg; 7 Programme in Emerging Infectious Diseases, Duke-NUS Medical School, Singapore 169857, Singapore

**Keywords:** polymyxins, pharmacokinetics, pharmacodynamics, polymyxin B nephrotoxicity

## Abstract

Population pharmacokinetic studies have suggested that high polymyxin B (PMB) doses (≥30,000 IU/kg/day) can improve bacterial kill in carbapenem-resistant Gram-negative bacteria (CR-GNB). We aim to describe the efficacy and nephrotoxicity of patients with CR-GNB infections prescribed high-dose PMB. A single-centre cohort study was conducted from 2013 to 2016 on septic patients with CR-GNB infection and prescribed high-dose PMB (~30,000 IU/kg/day) for ≥72 h. Study outcomes included 30-day mortality and acute kidney injury (AKI) development. Factors associated with AKI were identified using multivariable regression. Forty-three patients with 58 CR-GNB received high-dose PMB; 57/58 (98.3%) CR-GNB were susceptible to PMB. The median daily dose and duration of high-dose PMB were 32,051 IU/kg/day (IQR, 29,340–34,884 IU/kg/day) and 14 days (IQR, 7–28 days), respectively. Thirty-day mortality was observed in 7 (16.3%) patients. AKI was observed in 25 (58.1%) patients with a median onset of 8 days (IQR, 6–13 days). Higher daily PMB dose (aOR,1.01; 95% CI, 1.00–1.02) and higher number of concurrent nephrotoxins (aOR, 2.14; 95% CI; 1.03–4.45) were independently associated with AKI. We observed that a sizable proportion developed AKI in CR-GNB patients described high-dose PMB; hence, the potential benefits must be weighed against increased AKI risk. Concurrent nephrotoxins should be avoided to reduce nephrotoxicity.

## 1. Introduction

In the last decade, the global spread of carbapenem-resistant Gram negative bacteria (CR-GNB) has led to the reintroduction of polymyxin B [1,2]. The rekindled reliance on this once forsaken antibiotic has resulted in several questions regarding the clinical use of polymyxin B (e.g., doses in specific populations, toxicity profile) [1,3]. Unfortunately, as polymyxin B was developed before the introduction of modern drug developmental assays, data regarding its pharmacokinetic and pharmacodynamic properties are scarce and there is a paucity of information regarding its clinical use [1,3,4].

To date, there remains a lack of consensus on the most appropriate dosing regimen for polymyxin B [1,4]. Traditionally, polymyxin B has been dosed at 15,000 IU/kg/day to 25,000 IU/kg/day in accordance with the manufacturer’s recommendations, with dose reduction recommended in the setting of renal insufficiency [5]. However, a landmark population pharmacokinetics study has suggested that for organisms with polymyxin B MIC of ≤2 mg/L, higher polymyxin B doses may be necessary to achieve a ratio of the area under the concentration–time curve of the unbound antibiotic to the MIC (*f*AUC/MIC) of 20, which is a pharmacokinetic/pharmacodynamic (PK/PD) target that is most predictive for the in vivo activity of colistin but frequently extrapolated to polymyxin B [6]. Based on their findings, the authors suggested that in severe CR-GNB infections, regimens with a high daily dose (i.e., 30,000 IU/kg/day based on total body weight), with a loading dose of 25,000 IU/kg, should be considered [6]. The authors further suggested that, unlike colistimethate, polymyxin B doses should not be adjusted in the presence of renal impairment, as polymyxin B clearance appears to be largely non-renal in nature [6,7].

In light of the findings of the population pharmacokinetics study, several tertiary references now recommend the use of high-dose polymyxin B, especially in patients infected with organisms with polymyxin B MIC of ≤2 mg/L [8,9]. However, studies describing the outcomes of patients prescribed such high-dose polymyxin B regimens are limited. The purpose of this study is to describe the efficacy and nephrotoxicity outcomes in a cohort of septic patients with CR-GNB infections receiving high-dose polymyxin B. We additionally identified factors with acute kidney injury (AKI) development in these patients. 

## 2. Material and Methods

### 2.1. Study Setting and Population 

We conducted a retrospective cohort study in a 1700-bedded tertiary restructured Singapore hospital in accordance with the Strengthening the Reporting of Observational Studies in Epidemiology (STROBE) guidelines [10]. All patients admitted from July 2013 to December 2016 with microbiologic evidence of CR-GNB infection and sepsis (defined as the presence of life-threatening organ dysfunction caused by a dysregulated host response to infection) due to the CR-GNB infection and prescribed high-dose polymyxin B for ≥72 h for the CR-GNB infection were included [11]. High-dose polymyxin B was defined as polymyxin B dose of at least 30,000 IU/kg/day for ≥72 h during treatment [6]. To account for deviations due to the rounding of doses during prescribing, dose deviation within 5% of the stated target dose was permitted (i.e., patients with a polymyxin B dose of 28,500 IU/kg/day for ≥72 h were considered to be prescribed high-dose polymyxin B). If a patient received more than one polymyxin B course for separate CR-GNB infection episodes within a single admission, only the first polymyxin B course was assessed for eligibility. Patients were excluded if they were <21 years old, pregnant, lactating or administered high-dose polymyxin B for <72 h. 

### 2.2. Ethics

This study was approved by the SingHealth ethics review board (reference no: 2016/2681), and all research was conducted in accordance with the institutional standards. The need for informed consent was waived as this study was non-interventional in nature.

### 2.3. Data Collection

All patients were followed up until discharge or death. Data extracted from patients’ inpatient charts and electronic records included demographics, baseline comorbidities and age-adjusted Charlson comorbidity index, type of infection(s) and causative organisms, Acute Physiology and Chronic Health Evaluation II (APACHEII) score at the time of CR-GNB infection and high-dose polymyxin B initiation, renal insufficiency at time of high-dose polymyxin B initiation, details of polymyxin B use (loading dose, daily dose based on total body weight, duration of treatment and cumulative polymyxin B dose), concomitant infections and antibiotic use, concurrent nephrotoxic agents use and presence of source control for the CR-GNB infection [12,13]. Infections were defined according to the definition provided by the European Centre for Disease Control and Prevention [14]. Renal insufficiency at the time of high-dose polymyxin B initiation was defined as a creatinine clearance (CrCl) of <50 ml/min or the use of renal replacement therapy at the time of initiation [15]. Source control was defined as the removal of urinary or central venous catheters, drainage of intra-abdominal or deep-tissue collections or debridement of infected tissue. 

### 2.4. Study Outcomes

The efficacy outcomes were 30-day in-hospital all-cause and infection-related mortality, presence of clinical response and complete clinical cure, and presence of microbiological eradication (assessed only in bloodstream infections). Thirty-day in-hospital all-cause mortality and 30-day in-hospital infection-related mortality were respectively defined as all-cause and infection-related death that occurred within 30 days from the initiation of high-dose polymyxin B. Presence of clinical response was defined as a partial or complete resolution of infection symptoms (determined by the attending Infectious Diseases (ID) physician), while complete cure was defined as the complete resolution of infection symptoms. Microbiologic eradication was defined as the clearance of CR-GNB in all follow-up blood cultures. 

The nephrotoxicity outcomes included presence of AKI and time at risk to AKI during polymyxin B treatment and were assessed for all patients not receiving renal replacement therapy at the start of high-dose polymyxin B initiation. AKI due to high-dose polymyxin B was assessed in accordance with the RIFLE criteria and defined as attaining the injury tier (doubling of serum creatinine from baseline) or higher in the RIFLE criteria after ≥24 h from initiation of high-dose polymyxin B [16]. Time at risk to AKI due to high-dose polymyxin B was defined as time from initiation of high-dose polymyxin B to development of AKI. For those who did not develop AKI due to high-dose polymyxin B, time at risk was defined as time from initiation of high-dose polymyxin B to time of high-dose polymyxin B discontinuation. Serum creatinine levels and CrCl, which were routinely done in critically ill patients at least every 48 hours during hospital stay until discharge or death, were documented. CrCl was calculated using the Cockcroft–Gault equation, using adjusted body weight [17].

### 2.5. Microbiological Methods

The genus identity of all isolates was determined using VITEK GNI+ cards (bioMérieux, Hazelwood, MO, USA) by the hospital’s microbiology laboratory. CR-GNBs were defined as *Pseudomonas aeruginosa*, *Acinetobacter* spp. or Enterobacteriaceae that were not susceptible to carbapenems. Susceptibility to various antibiotics was determined using disk diffusion and interpreted in accordance with the Clinical and Laboratory Standards Institute (CLSI) guidelines [18]. MICs to meropenem, polymyxin B and tigecycline (only for *Acinetobacter* spp. isolates) were determined using E-test method (bioMérieux, Hazelwood, MO, USA), and susceptibility was defined as breakpoints provided by CLSI [18]. For Enterobacteriaceae, susceptibility to polymyxin B was defined as MIC ≤ 2 mg/L. 

### 2.6. Statistical Analysis

All data were analysed using Stata MP 15. Continuous data were presented as median and interquartile ranges (IQR), while categorical data were presented as numbers and percentages. To determine the factors associated with AKI in this study cohort, patients with and without AKI were compared using univariate logistic regression. Risk factors with *p* < 0.1 in the univariate analysis with clinical plausibility were included into a multivariable logistic regression model. The final model was selected based on clinical plausibility and minimisation of Akaike’s information criterion [19]. A final two-tailed *p* ≤ 0.05 was considered significant.

## 3. Results

### 3.1. Baseline and Infection Characteristics

From July 2013 to December 2016, 58 patients with sepsis received high-dose polymyxin B for ≥72 h. Fifteen patients were excluded as they did not have microbiologic evidence of CR-GNB infection, and 43 patients were included in the final analysis. The baseline demographics and clinical characteristics of the patients are described in Table 1. The median age of patients was 54 years (IQR, 33–66 years). The most common comorbidity was solid and haematological malignancy (32.6%). Only four (9.3%) patients had documented pre-existing chronic kidney disease; none of these patients were on chronic renal replacement therapy. The median Charlson comorbidity index was 3 (IQR, 0–4).

At the time of infection onset, the median APACHE II score was 17 (IQR, 11–22). The most sites of common CR-GNB infections were bloodstream infections (55.8%) and complicated skin and soft tissue infections (18.6%). A total of 58 CR-GNB were implicated, comprising 31 CR *Acinetobacter* spp. (53.4%), 13 CR *P. aeruginosa* (22.4%) and 14 CR Enterobacteriaceae (24.1%) (Table 2). Fifty-seven isolates (98.3%) were susceptible to polymyxin B; polymyxin B MICs ranged from 0.25 to 16 mg/l for CR-GNB isolates. 

### 3.2. Characteristics of Polymyxin B Use

The median time to initiation of high-dose polymyxin B from the onset of infection was 4 days (IQR, 2–5 days) (Table 1). In most patients (93.0%), polymyxin B was administered as part of a two- or three-antibiotic combination regimen, with polymyxin B plus carbapenem (57.5%) being the most common combination. Only 6 (14.0%) patients received a loading dose of polymyxin B. The median daily dose and duration of high-dose polymyxin B use was 32,051 IU/kg/day (IQR, 29,340–34,884 IU/kg/day) and 14 days (IQR, 7–28 days), respectively. Twelve (27.9%) patients received standard-dose polymyxin B (defined as polymyxin B dose of 15,000–25,000 IU/kg/day) for a median duration of 2 days (IQR, 2–4 days) prior to initiation of high-dose polymyxin B.

### 3.3. Efficacy Outcomes

A total of 7 (16.3%) patients died within 30 days from initiation of high-dose polymyxin B; of these, 3 (7.0%) were attributed to the CR-GNB infection. Presence of clinical response and complete clinical cure were observed in 32 (74.4%) and 26 (60.4%) patients, respectively. Microbiologic eradication was achieved in 23 (95.8%) out of the 24 patients with bloodstream infections. Other than one patient who already had a polymyxin B-resistant infection at infection onset, polymyxin B resistance was not observed during the follow-up period in the other patients. 

### 3.4. Nephrotoxicity Outcomes

**Figure 1:**
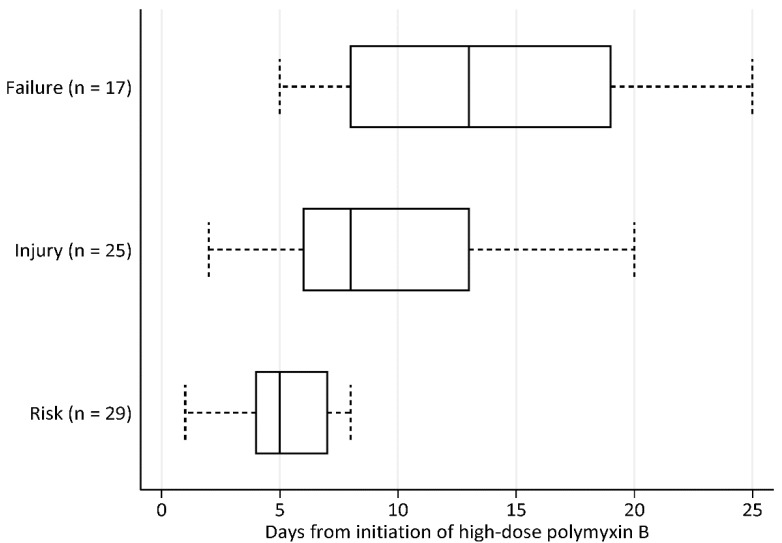
Days to (a) risk (*n* = 29), (b) injury (*n* = 25) and (c) failure (*n* = 17) based on the RIFLE criteria, from initiation of high-dose polymyxin B. The median times (IQR) to risk, injury and failure were 5 days (IQR, 4–7 days), 8 days (6–13 days) and 13 days (8–19 days), respectively.

**Figure 2:**
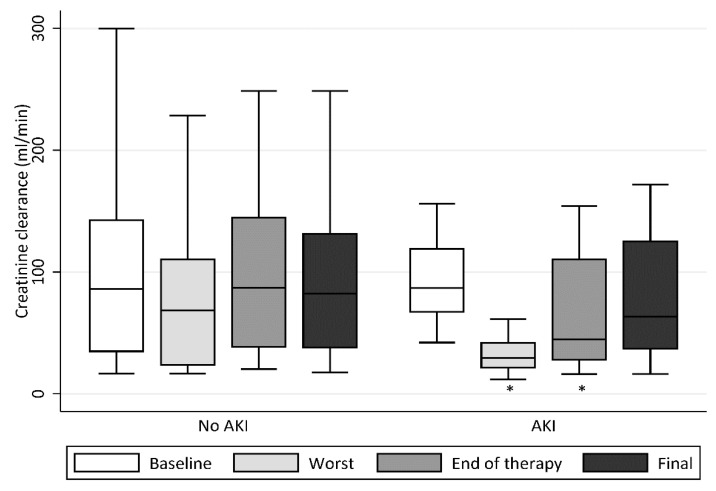
Estimated creatinine clearance values for patients with and without AKI during the start of high-dose polymyxin B therapy (baseline), lowest value during high-dose polymyxin B therapy (worst), end of high-dose polymyxin B therapy (end of therapy) and at discharge or death (final). * *p* < 0.05 compared to baseline creatinine clearance values.

We illustrated the time to onset of each stage of renal impairment in Figure 1. As shown, 25 (58.1%) patients developed AKI during high-dose polymyxin B therapy, with an onset time of 8 days (IQR, 6–13 days) from initiation of high-dose polymyxin B. Of these, 12 (36.0%) patients had polymyxin B doses reduced by their physician after onset of nephrotoxicity. Fourteen patients (56.0%) required renal replacement therapy during polymyxin B therapy. None of the patients developed AKI that progressed to end-stage renal disease due to high-dose polymyxin B. The temporal trends of CrCl levels of patients with and without AKI are shown in Figure 2. In patients without AKI, there was a nonsignificant reduction of CrCl levels observed during treatment compared to baseline (*p* = 0.31); this was followed by recovery of CrCl levels to baseline levels at the end of follow-up. In patients with AKI, we observed a significant reduction of CrCl levels during treatment compared to baseline levels (*p* < 0.01). The AKI appeared to subside upon discontinuation of polymyxin B with CrCl levels returning to near baseline level (CrCl levels observed at the end of follow-up in the patients with AKI were not significantly different from baseline, *p* = 0.18). A bivariate and multivariable analysis of patients who developed AKI is summarised in Table 3. In the bivariate analysis, higher daily doses of polymyxin B and number of concurrent nephrotoxins during polymyxin B therapy were significantly associated with the development of AKI. These factors remained significantly associated with AKI development in the multivariable analysis. 

## 4. Discussion

In this study, we described the outcomes of septic patients receiving high-dose polymyxin B for CR-GNB infections. While we observed promising mortality rates in our study when compared to historical cohorts, a sizable proportion of patients developed AKI during high-dose polymyxin B therapy [20,21,22]. The AKI appeared to subside upon discontinuation of polymyxin B, and none of the patients progressed to end-stage renal failure. Higher daily polymyxin B doses and increased number of concurrent nephrotoxins were independently associated with the development of AKI in patients on high-dose polymyxin B. 

There is renewed interest in polymyxin B in recent years. Compared to colistin, polymyxin B appears to have superior pharmacological properties [3,23,24]. Unlike colistin, which is administered as a prodrug, polymyxin B is administered in its active antibacterial form, which results in a rapid and reliable attainment of the plasma concentration of the active constituent upon administration [3,23,24]. The disposition of polymyxin B is minimally affected by renal function [6,7]. Hence, polymyxin B has substantially lower interindividual pharmacokinetic variability compared to colistin, and allows for dosing simplicity in patients with impaired or fluctuating renal function [6,23,24]. Despite its promising pharmacokinetic and pharmacodynamic properties, one of main shortcomings of polymyxin B is its propensity for nephrotoxicity. In a study by Azad et al., it has been proposed that the major pathways of polymyxin-induced nephrotoxicity were concentration-dependent and time-dependent [25]. Unfortunately, decreasing the daily doses of polymyxin B in hopes of avoiding nephrotoxicity is not a viable option, as it has been suggested that administering less than 15,000–25,000 IU/kg/day of polymyxin B will lead to subtherapeutic antibiotic exposure [4,6]. Such subtherapeutic exposure may have multiple detrimental effects, including compromising clinical outcomes due to inadequate drug exposure, and amplification of polymyxin B-resistant subpopulations [26,27].

In this study, we observed that more than half of the patients prescribed high-dose polymyxin B developed AKI during therapy, with a median onset of eight days. These observed AKI rates were higher than in clinical studies with patients receiving standard-dose polymyxin B but were comparable to the rates reported in a recent clinical study on patients with high-dose polymyxin B [15,20,28]. Among patients already receiving high polymyxin B doses, higher daily polymyxin B doses (median dose of polymyxin B in patients with AKI = 33,000 IU/kg/day versus median dose of polymyxin B in patients without AKI = 30,000 IU/kg/day) and concurrent nephrotoxin use were associated with increased risk of AKI development. Our findings corroborated the results from previous studies proposing that higher daily polymyxin B doses were associated with increased nephrotoxicity risk [15,28,29,30]. Interestingly, baseline creatinine clearance did not appear as a significant risk factor for AKI in our study, which is in contrast to the recent findings in a cohort study on high-dose polymyxin B by John et al. [21]. 

In our study cohort, most of the study patients were prescribed high-dose polymyxin B as part of a combination therapy. Loading doses for polymyxin B were not routinely prescribed by local physicians, which is in contrast to recommendations from current literature [8,9]. We observed a tendency for physicians to reduce polymyxin B doses upon the onset of nephrotoxicity, which we postulate may result in consequent subtherapeutic drug exposure in these patients, and further pharmacokinetic studies will be needed to explore this hypothesis. We observed promising in-hospital thirty-day all-cause mortality and high rates of microbiological eradication in patients with bloodstream infection among our study patients. These mortality rates appear to be lower compared to historical cohorts in previous clinical studies [20,21,22]. However, we acknowledge that the patient populations in different studies should not be subjected to direct comparison due to differences in study population characteristics, severity of infection and treatment regimens employed, and further comparative trials will be necessary to shed more light on the clinical utility of high-dose polymyxin B. In light of our study findings and considering current pharmacokinetics evidence on polymyxin B, we propose that the benefits of high-dose polymyxin B and the increased risk of AKI must be weighed against the high mortality associated with CR-GNB infections [6]. This is especially important in the treatment of septic patients, as treatment failure often equates to mortality [26]. If high-dose polymyxin B is employed in septic patients with CR-GNB infections, concurrent nephrotoxins should be avoided during high-dose polymyxin B use to reduce the risk of polymyxin B-induced AKI. A loading dose of 25,000 IU/kg should be considered to maximise the chances of achieving adequate polymyxin B exposure within the first day of polymyxin B therapy [6]. In addition, it must be kept in mind that exceedingly high doses of polymyxin B (i.e., >33,000 IU/kg/day) are associated with greater nephrotoxicity risk. 

There are a number of limitations to this study. Most importantly, as this is a noninterventional study, a substantial number of patients received standard-dose polymyxin B prior to the initiation of high-dose polymyxin B or had the polymyxin B dose reduced by their physician due to onset of nephrotoxicity. As a result, the efficacy of the high-dose polymyxin B in this study may have been attenuated by this dose reduction. Secondly, the serum polymyxin B concentrations in our study cohort were not measured, and we cannot conclude if the target *f*AUC/MIC of 20 has been achieved in our study population. Lastly, as mentioned, although we observed favourable clinical outcomes in our patients, we cannot associate the low rates of mortality to the high-dose polymyxin B regimen due to the lack of comparative data. Moving forward, comparative clinical studies between patients on high-dose and standard-dose polymyxin B will be highly desirable. 

## 5. Conclusions

Our study provided an insight into patient outcomes when high doses of polymyxin B are prescribed in patients with CR-GNB infections. While we await new and effective therapeutic options against CR-GNB, we should preserve the existing antibiotic armamentarium though appropriate and optimised prescribing. We conclude that when high-dose polymyxin B is employed, the potential benefits must be weighed against the increased risk of AKI. To minimise the risk of polymyxin B-induced AKI, concurrent nephrotoxins should be avoided. 

## Figures and Tables

**Table 1:** Baseline, infection and treatment characteristics of study cohort.

Variable *Median (IQR) or No. (%)*	All Patients (*n* = 43)
**Demographics and Admission Characteristics**	
Age (years)	54 (33–66)
Male gender	30 (69.8)
Length of hospital stay	55 (39–96)
Admission to ICU	12 (27.9)
Total body weight (kg)	60 (50–71)
**Comorbidities**	
Congestive heart failure	4 (9.3)
Chronic kidney disease	4 (9.3)
Diabetes mellitus	8 (18.6)
Solid and haematological malignancy	14 (32.6)
Age-adjusted Charlson comorbidity index	3 (0–4)
**Infection Characteristics and Clinical Presentation**	
Time to infection onset (days)	10 (4–28)
APACHE II score at infection onset	17 (11–22)
Presence of sepsis	43 (100.0)
Septic shock	38 (88.4)
**Types of Infection**	
Bloodstream infections	24 (55.8)
Complicated intra-abdominal infections	1 (2.3)
Complicated skin and soft tissue infections	8 (18.6)
Complicated urinary tract infections	3 (7.0)
Pneumonia	2 (4.7)
Multiple infection types	5 (11.3)
**Types of CR-GNB (Total No. of CR-GNB = 58) *^a^***	
*Acinetobacter* spp.	31 (53.5)
Enterobacteriaceae	14 (24.1)
*P. aeruginosa*	13 (22.4)
Patients with concurrent non-CR-GNB infections	29 (67.4)
**Characteristics of Treatment**	
APACHE II score at time of high-dose PMB initiation	21 (12–24)
Use of PMB loading dose (>25,000 IU/kg)	6 (14.0)
Daily high-dose PMB dose (IU/kg)	32 051 (29,340–34,884)
Duration of high-dose PMB (days)	14 (7–28)
Cumulative high-dose PMB (MIU)	24.5 (13.8–52.8)
Dose reduction or use of standard-dose PMB (≤25,000 IU/kg/day) prior to high-dose PMB	22 (51.6)
Overall average daily PMB dose (IU/kg) *^b^*	29 412 (28,070–33,751)
Overall cumulative PMB (MIU) *^b^*	27.0 (18.4–67.5)
Use of combination therapy	40 (93.0)
Presence of source control	25 (58.1)

*^a^* Ten patients had more infection due to ≥1 CR-GNB; *^b^* Values takes into account the dose reduction or use of standard dose polymyxin B prior to high-dose polymyxin B; NOTE: CR-GNB, carbapenem-resistant Gram-negative bacteria; ICU, intensive care unit; IU, international units; MIU, million international units; PMB, polymyxin B.

**Table 2:** Susceptibility profile and MICs of CR-GNB to various antibiotics.

Organisms	*Acinetobacter* spp. (*n* = 31)	*P. aeruginosa* (*n* = 13)	*Enterobacteriaceae* (*n* = 14)
**Susceptibility Profiles *^a^***			
Antimicrobial Agents	No. of non-susceptible isolates (%)	No. of non-susceptible isolates (%)	No. of non-susceptible isolates (%)
Ampicillin/sulbactam	31 (100)	ND	ND
Piperacillin/tazobactam	31 (100)	12 (92.3)	14 (100)
Cefepime	31 (100)	11 (84.6)	13 (92.9)
Meropenem	31 (100)	13 (100)	14 (100)
Aztreonam	ND	12 (92.3)	14 (100)
Ciprofloxacin	31 (100)	11 (84.6)	14 (100)
Tigecycline	14 (45.2)	ND	1 (7.1)
Gentamicin	29 (93.5)	12 (92.3)	11 (78.6)
Polymyxin B	0 (0)	0 (0)	1 (7.1)
**Minimum Inhibitory Concentrations**			
	MIC range (mg/l)	MIC range (mg/l)	MIC range (mg/l)
Meropenem	4—≥32	4—≥32	2—≥32
Polymyxin B	0.25—1	0.5—2	0.25—16

*^a^* Susceptibility was determined using disk diffusion defined as breakpoints provided by CLSI [18]; NOTE: MIC, minimum inhibitory concentrations; ND, not done.

**Table 3:** Factors potentially associated with acute kidney injury in patients with high-dose polymyxin B therapy.

Variable *Median (IQR) or No. (%)*	Univariate Analysis			Multivariable Analysis	
	No AKI (*n* = 18)	AKI (*n* = 25)	*p* Value	Adjusted OR (95% CI)	*p* Value
**Demographics and Infection Characteristics**					
Age (years)	55 (27–66)	54 (36–66)	0.89		
Male gender	12 (66.7)	18 (72.0)	0.71		
Age-adjusted Charlson comorbidity index	3 (1–6)	3 (0–4)	0.21		
APACHE II at infection onset	16 (12–22)	19 (11–21)	0.74		
APACHE II at high-dose PMB initiation	17 (12–22)	22 (15–24)	0.22		
Renal insufficiency at high-dose PMB initiation	5 (27.8)	3 (12.0)	0.20		
In-hospital 30-day all-cause mortality	3 (16.7)	5 (20.0)	0.78		
**Details of PMB dosing prior to time at risk *^a^***					
Duration of PMB (days)	12 (7–20)	12 (8–14)	0.68		
Overall average daily PMB dose (IU/kg)	30 273 (29 126–33 333)	33 708 (30 000–37 037)	0.04	1.01 (1.00–1.02)	0.04
Overall cumulative PMB (MIU)	20 (12–30)	23 (16–27)	0.95		
Use of combination therapy	17 (94.4)	23 (92.0)	0.76		
**Use of concomitant nephrotoxins**					
Diuretics	5 (27.8)	12 (48.0)	0.19		
Vancomycin	16 (88.9)	24 (96.0)	0.39		
Aminoglycosides	7 (38.9)	14 (56.0)	0.27		
Intravenous contrast	4 (22.2)	11 (44.0)	0.15		
Total number of nephrotoxins	2 (1–2)	3 (2–3)	0.04	2.14 (1.03–4.45)	0.04

*^a^* Time at risk was defined as the time from admission to development of AKI for patients who developed AKI due to high-dose PMB, and the time from admission to death or discharge for those who did not develop AKI due to high-dose PMB; NOTE: IU, international units; MIU, million international units; PMB, polymyxin B.
